# Effects of Micro-environmental pH of Liposome on Chemical Stability of Loaded Drug

**DOI:** 10.1186/s11671-017-2256-9

**Published:** 2017-08-23

**Authors:** Xiao-Ru Shao, Xue-Qin Wei, Shu Zhang, Na Fu, Yun-Feng Lin, Xiao-Xiao Cai, Qiang Peng

**Affiliations:** State Key Laboratory of Oral Diseases, West China Hospital of Stomatology, Sichuan University, No. 14, Block 3, Renmin Road South, Chengdu, 610041 China

**Keywords:** Liposomes, Nanoparticles, Drug delivery, Controlled release, Curcumin

## Abstract

Liposome is a promising carrier system for delivering bioactive molecules. However, the successful delivery of pH-sensitive molecules is still limited by the intrinsic instability of payloads in physiological environment. Herein, we developed a special liposome system that possesses an acidic micro-environment in the internal aqueous chamber to improve the chemical stability of pH-sensitive payloads. Curcumin-loaded liposomes (Cur-LPs) with varied internal pH values (pH 2.5, 5.0, or 7.4) were prepared. These Cur-LPs have similar particle size of 300 nm, comparable physical stabilities and analogous in vitro release profiles. Interestingly, the chemical stability of liposomal curcumin in 50% fetal bovine serum and its anticancer efficacy in vitro are both micro-environmental pH-dependent (Cur-LP-2.5 > Cur-LP-5.0 > Cur-LP-7.4). This serum stability still has space to be further enhanced to improve the applicability of Cur-LP. In conclusion, creating an acidic micro-environment in the internal chamber of liposome is feasible and efficient to improve the chemical stability of pH-sensitive payloads.

## Background

Liposome, an artificial membrane vehicle, has shown great potentials in drug delivery due to its drug loading capacity, biodegradability, and biocompatibility [1–4]. The classic liposome is similar with living cells in structure, typically consisting of a phospholipid bilayer and an aqueous inner chamber [5–7]. Due to this structure, liposome is able to solubilize the insoluble drug molecules and prevent the loaded drug from the harsh physiological environment [8–10]. In addition, the surface of liposome can be modified to prolong the blood circulation time and/or target specific tissues [11–15]. With these abovementioned advantages, various liposome systems have been clinically approved [8, 9, 16].

Although the delivery of many drugs has been improved by incorporation into liposome, the delivery of some pH-sensitive drugs is still limited by the instability of drug molecule itself in physiological environment (neutral pH values). Generally, liposome is prepared in a neutral buffer solution and thus the loaded drug molecules are also in a neutral environment after incorporation into liposome. Accordingly, those molecules which are only stable in acidic environment would be still instable even in the form of liposome. Therefore, development of a novel approach for enhancing the stability of pH-sensitive drugs is of great importance for successful delivery of these payloads by liposome.

**Fig. 1:**
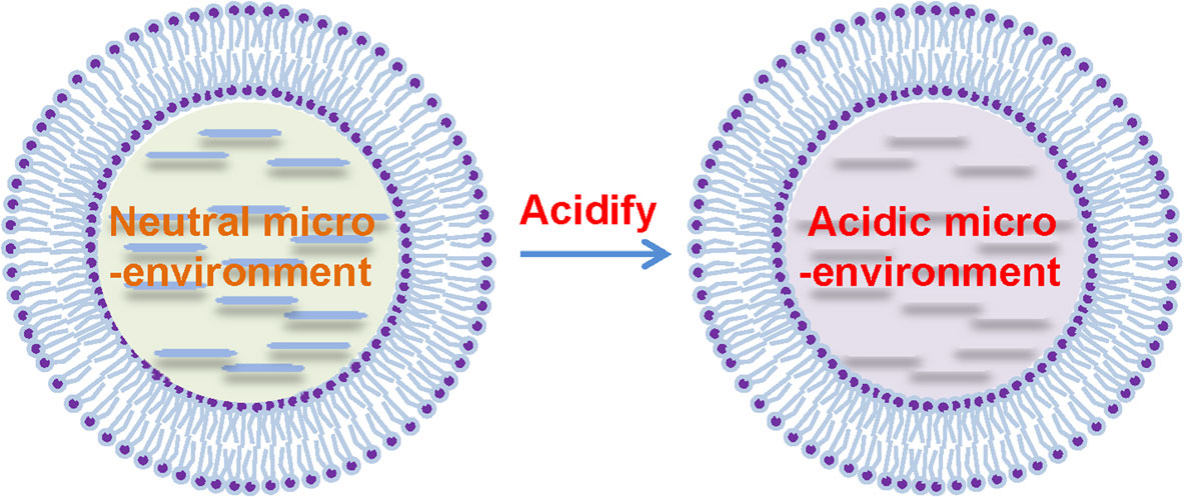
Schematics of the liposome with varied micro-environmental acidity in its inner aqueous chamber

As mentioned above, liposome has an aqueous space in its inner chamber, which can be used to provide drug payloads with an acidic micro-environment (Fig. 1). In this present work, we use curcumin as a model drug and aim to provide a novel approach for enhancing the chemical stability of drug molecules loaded in liposome. It is well known that curcumin is a lipophilic molecule and has been extensively used in food, medicines, and cosmetics due to its various bioactivities [17–21]. However, its delivery is highly limited by its insolubility and instability in biological fluids [22–25]. So far, it is yet to fulfill its clinical promise in part due to pH-mediated instability [26]. Therefore, curcumin is a suitable model drug for this work.

## Methods

### Materials

Phospholipids (soybean lecithin for injection use) were purchased from Shanghai Tai-Wei Pharmaceutical Co., Ltd., (Shanghai, China). Cholesterol was obtained from Amresco (Solon, OH, USA). Poloxamer 188 (F68) was kindly donated by BASF (China) Co., Ltd., (Shanghai, China). Curcumin was supplied by Sigma (St. Louis, MO, USA). Fetal bovine serum (FBS) was purchased from HyClone (Logan, UT, USA). All other chemical reagents used in this study were of analytical grade or better.

### Preparation of Curcumin-Loaded Liposomes (Cur-LPs)

The liposomes with varied micro-environmental pH values were prepared using evaporation method according to previous works with some modifications [27, 28]. Briefly, phospholipids (75 mg) and cholesterol (5 mg) were dissolved in 0.5 ml ethanol containing 2 mg/ml curcumin. The ethanol solution was mixed with 5 ml 0.001 M PBS containing 1% (*w*/*v*) F68 that served as a surfactant to narrow the size distribution. After magnetically stirring for 1 min (constant temperature magnetic mixer, DF-101S, Zhengzhou Greatwall Scientific Industrial and Trade Co., Ltd., Zhengzhou, China), the resultant emulsion was evaporated under vacuum and dark for 30 min at 35 °C to remove ethanol. The acidity in the inner chamber of Cur-LP was adjusted via using PBS with varied pH values of 2.5, 5.0, or 7.4 during preparation. The resultant suspension was centrifuged at a low speed (3000 rpm, 5 min) to precipitate free curcumin. The supernatant was then centrifuged at a high speed (16 krpm, 10 min), and the pellets were re-suspended in PBS (pH 7.4) before further use. This procedure provided these LPs with an identical external environment. The obtained liposomes with different micro-environmental pH values were presented as Cur-LP-2.5, Cur-LP-5.0, and Cur-LP-7.4, respectively. Blank liposomes were also fabricated as above.

### Characterization of Liposome

The hydrodynamic size, size distribution, and zeta potential are the three basic parameters for liposome systems. The size and zeta potential of LP were determined by dynamic light scattering (DLS) and electrophoretic light scattering (ELS), respectively, using ZetasizerNano ZS90 (Malvern Instruments Ltd., Malvern, UK) at 25 °C [29]. The measurement cycle was automatically determined by the instrument system. The particle size was presented by intensity distribution, and the size distribution was evaluated by polydispersity index (PDI).

### Encapsulation Efficiency (EE) Determination

EE, an important parameter for quality control, is of great significance in developing liposome-based delivery systems. The EE determination was based on the high speed centrifugation method. Briefly, 100 μl Cur-LPs was centrifuged at the low speed (3000 rpm, 5 min) to precipitate non-dissolved free curcumin, and 50 μl supernatant was subjected to high-speed centrifugation (16 krpm, 10 min) to separate Cur-LPs from the tiny dissolved curcumin. The pellets were re-suspended in 500 μl PBS (i.e., 10-fold dilution), an aliquot of 10 μl of which was mixed with 300 μl ethanol by vortex and sonication for 30 s. The fluorescent intensity of curcumin in the resultant solution was determined (excitation wavelength (Ex), 458 nm; emission wavelength (Em), 548 nm) and presented as *F*_*e*_, i.e., the fluorescent intensity of encapsulated curcumin. Another 50 μl of fresh Cur-LP containing encapsulated and free curcumin was also diluted by 10-fold with PBS, and 10 μl of the diluted solution were mixed with 300 μl ethanol. The fluorescent intensity of the resultant solution was measured and presented as *F*_*t*_, i.e., the fluorescent intensity of total curcumin. The EE was therefore calculated with the following equation: EE = *F*_*e*_/*F*_t_.

### Scanning Electron Microscopy (SEM)

The morphology of LP was observed by the scanning electron microscopy (SEM, INSPECT F, FEI, Netherlands) [30]. Briefly, the LP suspension was 100-fold diluted with distilled water, and one drop of the diluted suspension was placed on a clean glass sheet. After air-drying, the sample was coated with gold right before SEM.

### Physical Stability of Liposomes

Physical stability is a very meaningful parameter for storage and transportation of a colloidal system. The physical stability of liposome was presented by colloidal stability and investigated according to a previous method [31]. Briefly, 100 μl of LP was added to tubes and kept at 37 °C. At different time intervals, the LP size was measured and compared to the initial size so as to indicate the thermodynamic stability. In addition, another 300 μl of LP were also added to tubes and kept at 37 °C. At the same time intervals, 100 μl of the upper layer liquid were collected. The transmittance of the collected specimens was measured at 550 nm and compared to the initial value so as to indicate the kinetic stability.

### In Vitro Release

The release profile of liposome plays an important role in predicting in vivo fate and efficacy of liposome. The in vitro release of curcumin from Cur-LP was studied using the dynamic dialysis method [32]. Briefly, 1 ml of each Cur-LP was added into a dialysis bag (molecular weight cutoff, 10 kD), which was used to retain liposome but keep the released curcumin molecules permeable. The specimen-loaded dialysis bag was soaked in 4 ml release medium (0.001 M PBS containing 0.1% Tween 80, pH 7.4), and the release study was conducted away from light (37 °C, 100 rpm). At each fixed time interval, the release medium was collected and replaced with 4 ml fresh medium so as to simulate sink conditions. The collected medium was diluted to 5 ml with PBS and further diluted by 15-fold with ethanol. The curcumin in the resultant solution was quantified by fluorescence spectrophotometry (Ex 458 nm, Em 548 nm). In addition, curcumin powder was dissolved in the above release medium, and the release of curcumin solution was conducted at pH 7.4 to investigate whether the dialysis bag would retain curcumin molecules.

### Chemical Stability of Liposomal Curcumin

Chemical stability is a key parameter for predicting drug metabolism, efficacy, and toxicity. The chemical stability of Cur-LPs was examined in 50% FBS. Briefly, 100 μl of Cur-LPs were diluted by 10-fold with PBS (pH 7.4) and then mixed with 1 ml FBS. The specimens were shaken on a horizontal shaker away from light (37 °C, 100 rpm). At fixed time intervals, an aliquot of 10 μl of specimen was collected and mixed with 300 μl ethanol immediately followed by centrifugation (16 krpm, 5 min). The remained curcumin in the supernatant was quantified as above.

### In Vitro Anticancer Efficacy

The preliminary anticancer efficacy of the three Cur-LPs was investigated using human liver hepatocellular carcinoma HepG2 cells. Briefly, HepG2 cells were plated onto the 96-well cell culture plates at a density of 10,000 cells per well and cultured under standard conditions (37 °C/5% CO_2_) for 24 h in PRIM-1640 culture medium supplemented with 10% FBS. Subsequently, the culture medium was removed and cells were washed with PBS. The Cur-LPs were diluted in serum-free culture medium (4 μg/ml curcumin) and added to cells, followed by continuous incubation for 1 and 3 days at 37 °C. The OD value of viable cells was measured by cck-8 assay. The cells treated with blank culture medium served as control and cell viability (%) was the OD value percentage of specimens relative to control.

### Statistics

All the data are presented as mean ± sd (standard deviations). The differences between two groups, analyzed by the Student’s *t* test, were considered to be statistically significant when the *p* value was less than 0.05.

## Results and Discussion

### Characterization of Liposome

The micro-environmental pH of liposome refers to the acidity in the inner aqueous chamber of liposome (Fig. 1), which is different from the pH in the external environment. In this work, the external environmental pH of all liposome suspensions was 7.4 unless otherwise stated.

**Fig. 2:**
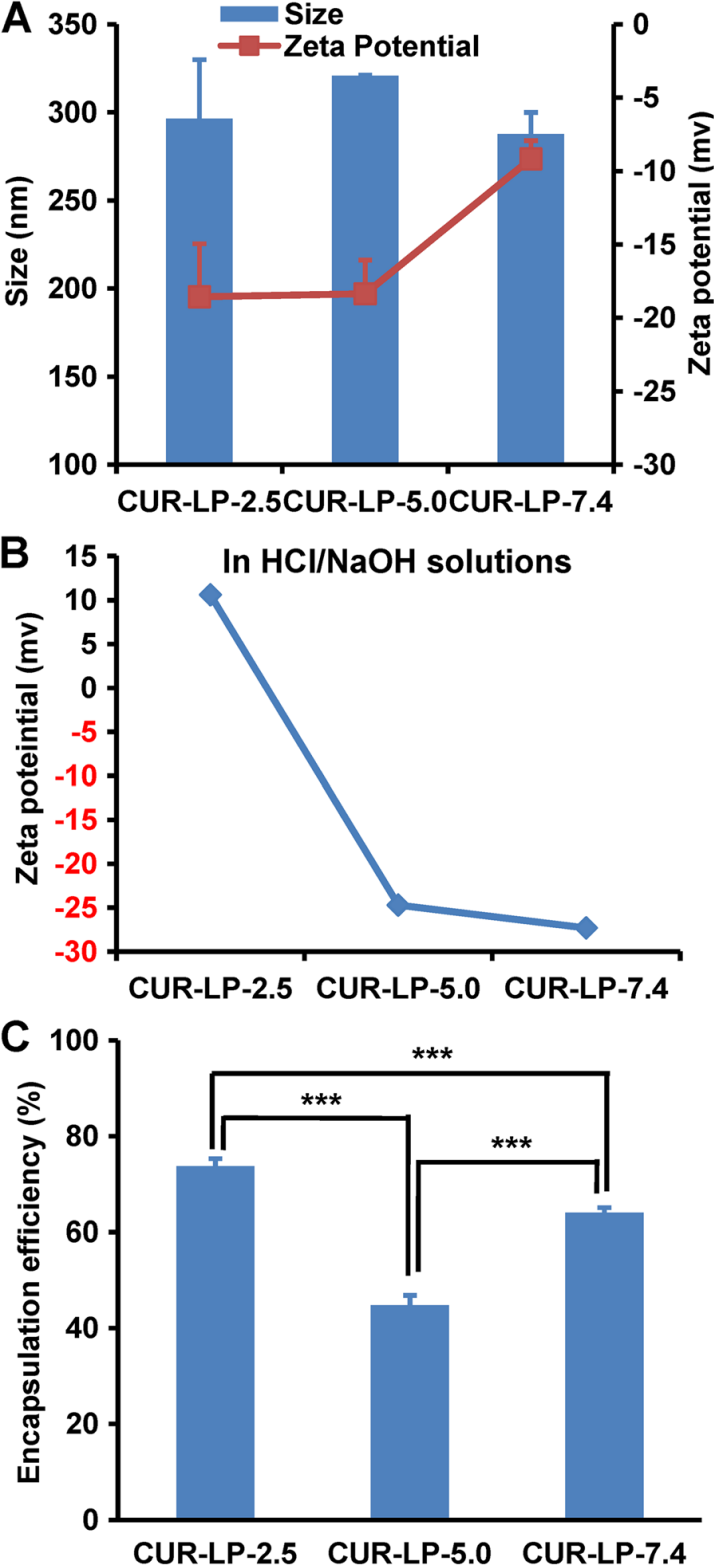
Physicochemical characterization of liposomes. **a** Hydrodynamic size and zeta potential of Cur-LPs fabricated in PBS with pH 2.5, 5.0, and 7.4, respectively. **b** Zeta potential of Cur-LPs fabricated in HCl/NaOH solutions with pH 2.5, 5.0, and 7.4, respectively. **c** Encapsulation efficiency of Cur-LPs prepared in PBS. Data presented as mean ± sd (*n* = 3). Statistical significance between groups: ***p < 0.001

Particle size, zeta potential, and encapsulation efficiency (EE) are important parameters for quality control of liposome. The size of three Cur-LPs was similar to each other (around 300 nm, Fig. 2a). The PDI of each formulation was lower than 0.2, indicating a narrow size distribution. Interestingly, the negative zeta potential of Cur-LP-7.4 (−9 mV) is significantly lower than that of the other two Cur-LPs (~−18 mV). Usually, the negative zeta potential would decrease and even convert to positive value with decreasing the pH of disperse phase due to the increase of H^+^ concentration. We indeed observed this phenomenon when preparing Cur-LPs in non-buffer HCl/NaOH solutions with pH of 2.5, 5.0, and 7.4 (Fig. 2b). In the case of PBS, however, the existence of PO_4_^3−^, HPO_4_^2−^, and/or H_2_PO_4_^−^ and their interaction with LP may lead to more complicated situations and different results. It is well recognized that zeta potential plays key roles in maintaining the colloidal stability of nano-scaled suspension. In general, a higher absolute value of zeta potential leads to a more stable colloidal suspension system.

EE is of concern during liposome development. Usually, increasing EE is important for reducing cost and enhancing efficacy. In this work, the EE of Cur-LP-2.5 is 74% (Fig. 2c), which is the highest among Cur-LP-5.0 (45%) and Cur-LP-7.4 (64%), indicating that Cur-LP-2.5 is the best formulation for delivering curcumin from the point of EE. The reasons for the variety in EE at different pH values are not very clear but may be related to the solubility of curcumin which is soluble in alkali or extremely acidic solvents [33].

**Fig. 3:**
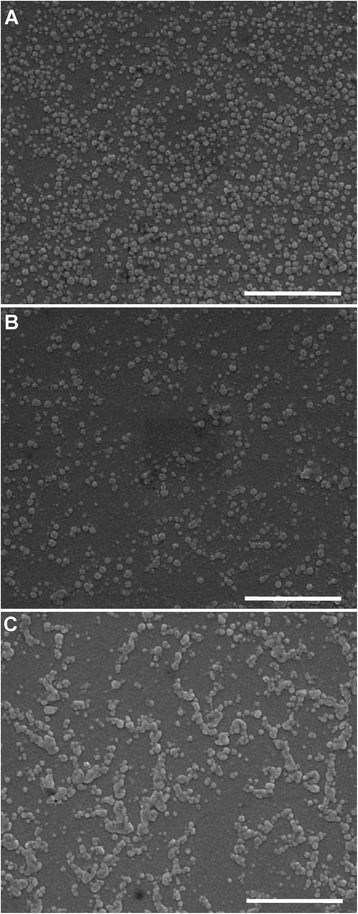
SEM images of liposome with micro-environmental pH of **a** 2.5, **b** 5.0, and **c** 7.4. *Scale bar*, 1 μm

The morphology of liposomes examined by SEM is shown in Fig. 3. The particles of LP-2.5 (Fig. 3a) and LP-5.0 (Fig. 3b) are spherical in shape and have a uniform particle distribution. The LP-7.4 also shows a sphere-like shape, but adhesion among particles can be clearly observed (Fig. 3c), indicating that the drying process during SEM specimen preparation would lead to aggregation of LP-7.4. This may be due to the relatively low absolute value of zeta potential of LP-7.4 (Fig. 2a). Additionally, the particle size measured by SEM is smaller than the hydrodynamic size measured by DLS, which is due to the loss of hydration shell of liposome after drying process for SEM.

### Physical Stability of Liposome

Liposome is a colloidal system and its physical stability can be presented by colloidal stability, which has substantial impacts on liposome storage and further applications [34, 35]. Particle aggregation (thermodynamic instability) and sedimentation (kinetic instability) are the two essential aspects of colloidal instability. Aggregation leads to a larger apparent size and sedimentation leads to changes in transmittance of suspension. More importantly, size increase can directly affect the efficacy of nano-systems since particle size has been shown to have great impacts on cellular uptake, cytotoxicity, pharmacokinetic profile, and tissue distribution [36, 37].

**Fig. 4:**
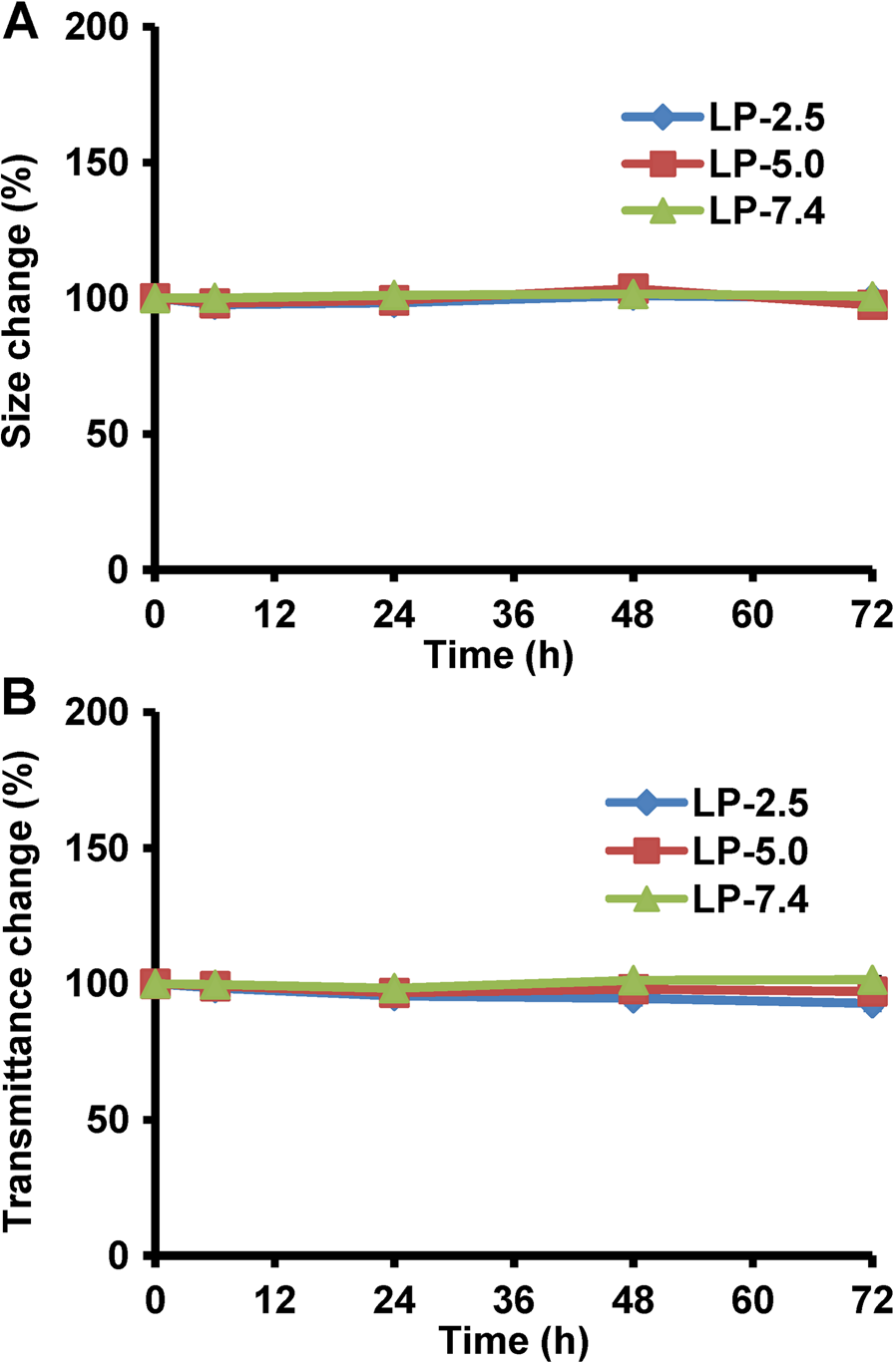
Physical stability of liposomes with varied micro-environmental pH values (pH 2.5, 5.0, and 7.4). **a** Thermodynamic stability indicating particles aggregation. **b** Kinetic stability indicating particle sedimentation. The data is presented as mean ± sd (*n* = 3)

Here, we examined the aggregation and sedimentation properties of three liposome systems to indicate their thermodynamic and kinetic stability, respectively. As shown in Fig. 4a, the three LPs showed no substantial changes in hydrodynamic size within 72 h, indicating that all these LPs have a very high thermodynamic stability. Meanwhile, the transmittance change of all the three LPs was less than 10% (Fig. 4b), indicating little particle sedimentation and thus a high kinetic stability. These results suggest that the three LPs have an excellent colloidal stability within 72 h, and the micro-environmental pH has no influence on physical stability of liposome.

### In Vitro Release

**Fig. 5:**
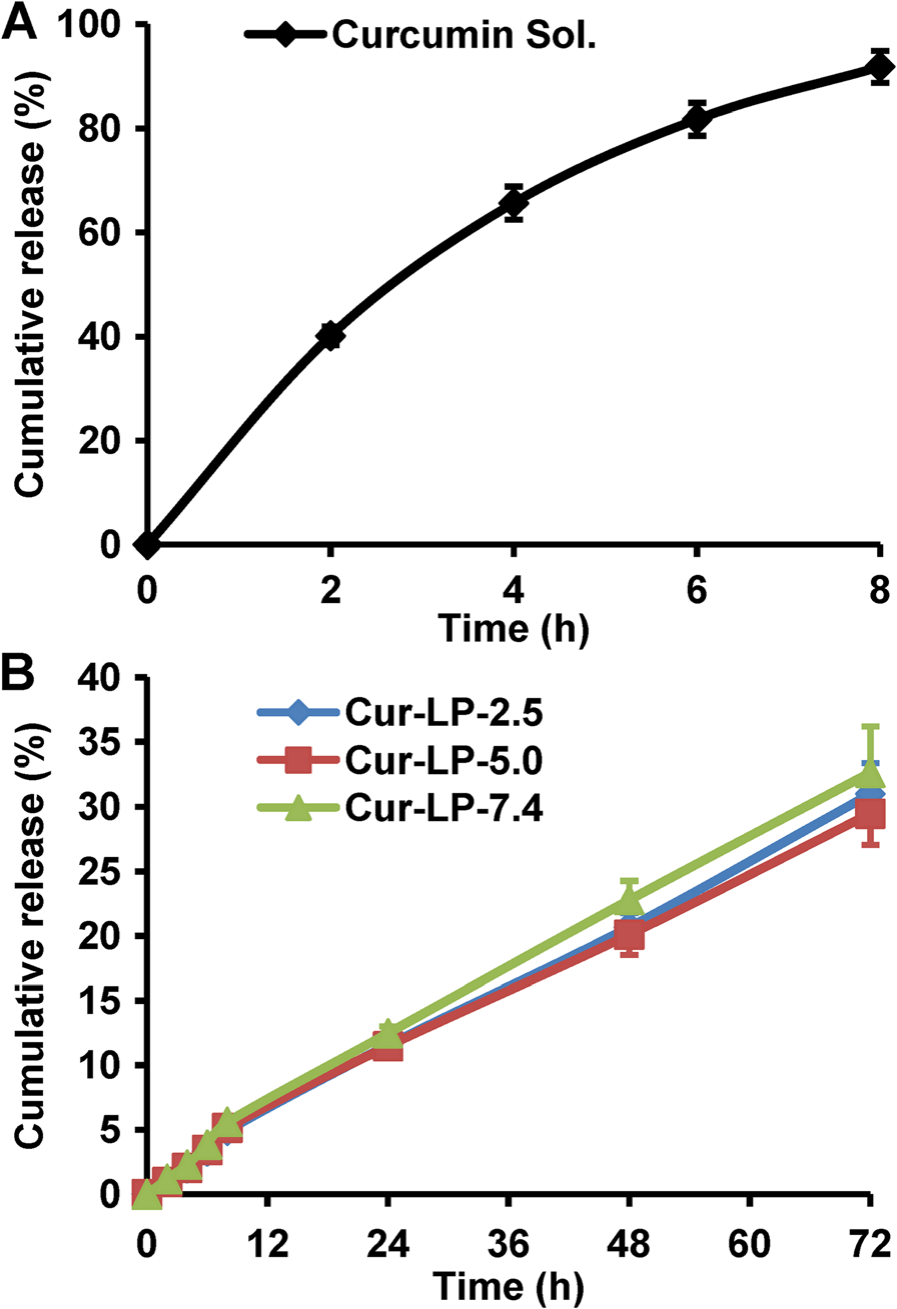
In vitro release profiles of different curcumin formulations in PBS (pH 7.4). **a** Curcumin solution, in which curcumin was dissolved in PBS containing 0.1% Tween 80 (pH 7.4). **b** Cur-LPs with varied micro-environmental pH of 2.5, 5.0, and 7.4, respectively. The data is presented as mean ± sd (*n* = 3)

Drug release profile from liposome is usually examined to evaluate formulation quality, provide reference for dosage regimen, and predict the effectiveness in vivo. In general, almost all liposomal systems have a sustained drug release property. Here, we examined the in vitro release behavior of three Cur-LPs in PBS (pH 7.4). Meanwhile, the release of curcumin solution was also examined so as to confirm whether the dialysis membrane would affect curcumin diffusion. As shown in Fig. 5a, curcumin was released very fast from its solution (>80% at 6 h), indicating that the dialysis bag had no effect on curcumin diffusion. In contrast to the rapid release of curcumin solution, all the Cur-LPs showed an obvious sustained release property (Fig. 5b), and the release profiles were very similar to each other, indicating that micro-environmental pH had no significant effect on curcumin release speed. In detail, curcumin was released a little faster in the first 8 h due probably to the initial burst release (the cumulative release percentage was around 5%). After 8 h, curcumin was released a little slower and the cumulative release percentage was ~30% within 72 h. It is assumed that the release speed in vivo or in the presence of serum would be substantially faster due partially to the metabolism of lipid.

Interestingly, the release profiles of all the three Cur-LPs are close to straight lines. Therefore, the linear fitting to the three release profiles was performed. As shown in Table 1, all these profiles showed very good linearity with fitting degree higher than 0.99 (regression equations are also displayed), suggesting that the release of Cur-LPs fitted to zero-order kinetics. In other similar studies, the release of curcumin from liposome was found to be non-linear [38, 39]. From the point view of drug research and development, zero-order release kinetics is the most ideal release profile because it provides a constant drug release rate and thus is able to maintain therapeutic effect for a long time, decrease administration times, and reduce side effects. Therefore, the LPs prepared in this work may be promising carriers for controlled drug delivery.

**Table 1:** Linear fitting of the in vitro release profiles of Cur-LPs with varied micro-environmental pH values

Liposomes	Regression equation	Fitting degree (*r*)
Cur-LP-2.5	*y* = 0.424x + 0.608	0.9985
Cur-LP-5.0	*y* = 0.403x + 0.800	0.9975
Cur-LP-7.4	*y* = 0.449x + 0.885	0.9980

### Effect of Micro-environmental pH on Chemical Stability of Cur-LP

**Fig. 6:**
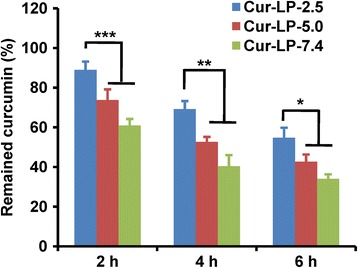
Chemical stability of liposomal curcumin (Cur-LPs) with varied micro-environmental pH values (pH 2.5, 5.0, and 7.4). The stability was examined by quantifying the remained curcumin after incubating Cur-LPs with 50% FBS. The data is presented as mean ± sd (*n* = 3). Statistical significance between groups: ***p < 0.001, **p < 0.01, *p < 0.05

The chemical stability of liposomal curcumin in FBS is shown in Fig. 6. After incubation for 2 h, 89% curcumin remained for Cur-LP-2.5, significantly higher than 74% for Cur-LP-5.0 and 61% for Cur-LP-7.4 (*p* < 0.001). At 4 h post-incubation, 69% curcumin remained for Cur-LP-2.5, significantly higher than 53% for Cur-LP-5.0 and 40% for Cur-LP-7.4 (*p* < 0.01). At 6 h post-incubation, 55% curcumin remained for Cur-LP-2.5, still significantly higher than 43% for Cur-LP-5.0 and 34% for Cur-LP-7.4 (*p* < 0.05). It is clear that the chemical stability of Cur-LPs is micro-environmental pH-dependent: Cur-LP-2.5 > Cur-LP-5.0 > Cur-LP-7.4. This pH-dependent chemical stability of Cur-LP is consistent with another work, which showed the pH-dependent stability of free curcumin [26]. The in vitro release was performed in serum-free medium, and the cumulative release could be 30% at 72 h. However, the chemical stability study was performed in serum-containing solution, in which serum enzymes could degrade the released curcumin and also break liposome and thus degrade the unreleased curcumin. This is the reason why 30% curcumin was released at 72 h in the in vitro release study but only 55% remained at 6 h for Cur-LP-2.5 in the serum stability study.

Liposomes consist of two parts in structure: one is the hydrophobic lipid bilayer and the other is the hydrophilic inner aqueous chamber. It is easy to understand that a pH-sensitive hydrophilic drug would be located in the inner aqueous chamber, and its stability would be significantly affected by the micro-environmental pH in the aqueous chamber, where the buffering volume and buffering capacity would be much higher than that in the lipid bilayer. In contrast, curcumin is a hydrophobic molecule and would be located in the lipid bilayer. For this reason, it is quite interesting to find the micro-environmental pH-dependent chemical stability of liposomal curcumin. It is assumed that the space in lipid bilayer would not be absolutely anhydrous although it is hydrophobic. As we know, the living cell membrane is not absolutely anhydrous in its lipid bilayer. Instead, it contains a certain small volume of aqueous solution for transport of water-soluble molecules and ions. Likewise, a certain small volume of buffer solution with the same components as the inner chamber would also exist in the hydrophobic lipid bilayer after successful preparation of liposome. Thus, the hydrophobic drug located in the lipid bilayer can be directly affected by the micro-environmental pH of liposome. In addition, the acidic micro-environment may reduce the activities of some enzymes which show the best activity in normal physiological condition. This also contributes to the higher chemical stability of liposomal curcumin in the lower micro-environmental pH. It has been reported that liposomes composed of egg phosphatidylcholine (EPC) rapidly lost their internal pH-gradient in buffer (pH 7.4), and the pH-gradient maintaining ability was substantially enhanced by substituting EPC (phase transition temperature (*T*_*m*_) ≈ −5 °C) with the high *T*_*m*_ (41 °C) lipid DPPC (dipalmitoyl phosphatidylcholine) and by addition of cholesterol [40]. In this present work, the liposome is composed of soybean lecithin (*T*_*m*_ is around 238.2 °C [41]) and cholesterol. Hence, the micro-environmental pH-gradient of liposomes prepared in this work can be expected to maintain for a long period. This is a strong support to the results and assumptions shown above.

### In Vitro Anticancer Efficacy

**Fig. 7:**
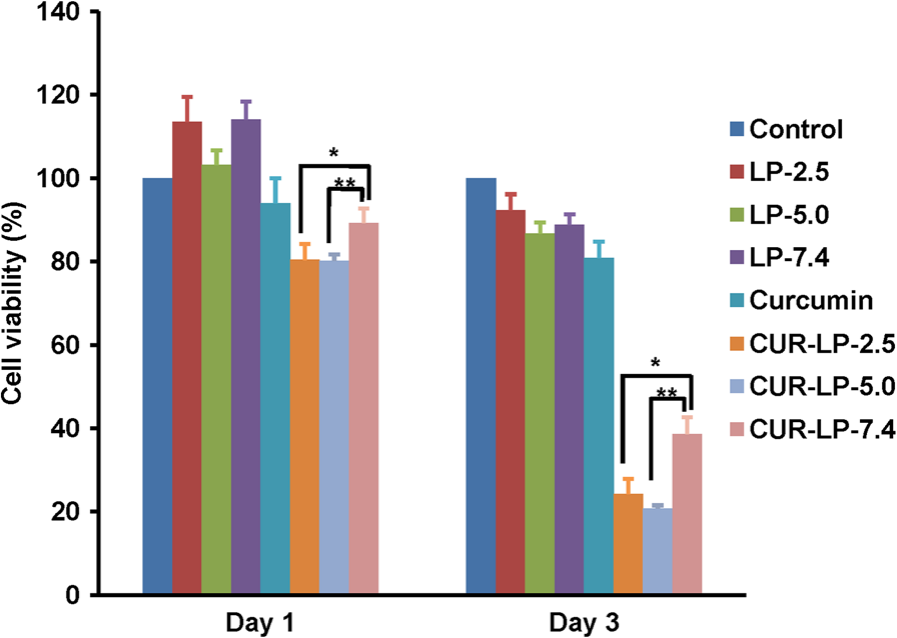
Anticancer efficacy of liposomal curcumin with varied micro-environmental pH (2.5, 5.0, and 7.4). The viability of HepG2 cells at days 1 and 3 after treatment by blank LPs, free curcumin, and Cur-LPs was examined by cck-8 assay. The cells treated by serum-contained blank culture medium served as control. The data is presented as mean ± sd (*n* = 3). Statistical significance between groups: **p < 0.01, *p < 0.05

We have demonstrated the micro-environmental pH-dependent chemical stability of liposomal curcumin above. Here, we conducted a preliminary in vitro study to investigate the anticancer efficacy of these liposomal curcumin. Interestingly, the blank LPs could enhance the cell growth at day 1 and maintain this function to some extent till day 3 in comparison to the control group (Fig. 7). This indicates that blank LPs may provide nutrition to cells, which is consistent with our previous report [27]. The free curcumin showed little anticancer efficacy due to its quite limited solubility. In contrast, Cur-LPs demonstrated significant anticancer efficacy in a micro-environmental pH-dependent manner. After treatment for 1 day, Cur-LP-2.5 and Cur-LP-5.0 showed a significantly stronger ability to inhibit HepG2 cell growth than Cur-LP-7.4 (the cell viability was 80% for Cur-LP-2.5 and Cur-LP-5.0, and 90% for Cur-LP-7.4). At day 3 post-treatment, the cell viability decreased substantially, and Cur-LP-2.5 and Cur-LP-5.0 showed comparable anticancer efficacy and significantly higher than Cur-LP-7.4. The cell viability was 24% for Cur-LP-2.5 (*p* < 0.05 vs Cur-LP-7.4), 21% for Cur-LP-5.0 (*p* < 0.01 vs Cur-LP-7.4), and 39% for Cur-LP-7.4. These results indicate that the anticancer efficacy of liposomal curcumin is micro-environmental pH- and time-dependent. In consideration of the higher EE and chemical stability of Cur-LP-2.5 than Cur-LP-5.0, liposome with micro-environmental pH of 2.5 would have the greatest potential for practical application.

## Conclusions

Liposome, as a widely used drug delivery system, is capable of improving the solubility of water-insoluble drugs, protecting the drug payloads from the harsh physiological environment and delivering the payloads to a targeted tissue. However, the delivery of pH-sensitive drugs is still limited by their natural instability in physiological conditions (neutral environment). In this present work, we propose a novel approach for enhancing the chemical stability of pH-sensitive drug payloads by regulating the micro-environmental acidity of liposome. The findings show that the chemical stability and in vitro efficacy of the model pH-sensitive drug curcumin is significantly enhanced by acidifying the micro-environment of liposome. In conclusion, regulation of micro-environmental pH of liposome is feasible to enhance the chemical stability of pH-sensitive drug payloads, even for the hydrophobic drugs which are located in the lipid bilayer.
